# The A-to-Z factors associated with cognitive impairment. Results of the DeCo study

**DOI:** 10.3389/fpsyg.2023.1152527

**Published:** 2023-06-20

**Authors:** María Gil-Peinado, Mónica Alacreu, Hernán Ramos, José Sendra-Lillo, Cristina García, Gemma García-Lluch, Teresa Lopez de Coca, Marta Sala, Lucrecia Moreno

**Affiliations:** ^1^Cátedra DeCo MICOF-CEU UCH, Universidad Cardenal Herrera-CEU, Valencia, Spain; ^2^Muy Ilustre Colegio Oficial de Farmacéuticos, Valencia, Spain; ^3^Department of Mathematics, Physics and Technological Sciences, Universidad CEU Cardenal Herrera, Valencia, Spain; ^4^Department of Pharmacy, Universidad Cardenal Herrera-CEU, CEU Universities, Valencia, Spain

**Keywords:** dementia, cognitive impairment, risk factors, protective factors, prevention, screening

## Abstract

**Introduction:**

Cognitive impairment (CI) is known to be mediated by several risk and protective factors, many of which are potentially modifiable. Therefore, it is important to have up-to-date studies that address a standard assessment of psychosocial, clinical and lifestyle variables.

**Materials and methods:**

We conducted a cross-sectional observational study, with a 24-month timeframe, to estimate the relationship between risk and protective factors associated with dementia, according to the A-to-Z Dementia Knowledge. Participants were considered at CI risk if they tested positive for at least one of three validated CI screening tests: The Memory Impairment Screening, Short Portable Mental State Questionnaire, and Semantic Verbal Fluency. The A-to-Z data Collection included Mediterranean Diet Adherence Screener and Geriatric Depression Scale.

**Results:**

The estimated prevalence of CI was 22.6% in a sample of 709 patients with an average of 69.3±10.3 years. The risk factors gradually associated with cognitive decline were hypertension, loneliness, and depression. In contrast, the protective factors gradually associated with less cognitive decline were internet use, reading, and intellectually stimulating jobs. Finally, living alone, having diabetes, taking benzodiazepines, and sleeping more than 9 h were statistically significant associated with CI, whereas to do memory training or a family history of dementia was characteristic of patients without CI.

**Conclusion:**

A joint assessment of the influence of psychosocial, clinical, and lifestyle-related factors is needed to develop dementia prevention strategies.

## 1. Introduction

According to the 2021 World Alzheimer Report, “dementia”, a major neurocognitive disorder, is not a specific disease, but a collection of symptoms resulting from an underlying condition. Dementia significantly affects memory, behavior, thinking, and social abilities severely enough to interfere with one's activities of daily living and social autonomy (Prince et al., 2016; Alzheimer Disease International, 2021).

In 2020, the National Institute on Aging and the Alzheimer's Association published a toolkit with six distinct stages of Alzheimer's disease (AD) (Jack et al., 2018). The first stage of the disease is characterized by the absence of subjective or objective evidence of cognitive impairment (CI) or behavioral disturbances. The second transitional stage includes people who exhibit subjective memory complaints (SMC), subtle objective impairment, or mild behavioral symptoms. These two are the so-called “prodromal stages”, while the third phase is the so-called “mild cognitive impairment” (MCI). Finally, stages 4 to 6 represent different clinical periods of dementia: mild, moderate, and severe (Jack et al., 2018; Jessen et al., 2020).

MCI is a syndrome defined as a cognitive decline that exceeds what is expected for an individual's age and education level but without notably interfering with daily life activities (Lopez et al., 1999). It is characterized by objectively measured CI using validated neuropsychological tests (Jessen et al., 2020). Patients with CI are at a higher risk of developing AD or other types of dementia compared to the general population (Petersen, 2006).

Dementia is a progressive neurodegenerative disease that can manifest up to 20 years before diagnosis. CI stands out as a prelude to the pathology, characterized by a decline in cognitive abilities when the patient does not meet the criteria for dementia diagnosis (Jessen et al., 2020). Thus, early detection of CI is essential as it is during this preclinical phase where a more significant benefit can be expected with disease-modifying or slowing therapies (Ramos et al., 2021b).

SMC is defined as the subjective perception of a decline cognitive abilities compared to previous levels of functioning in individuals with normal cognition. Evidence suggests that SMC may represent the first preclinical manifestation of AD (Warren et al., 2022). Nowadays, There is a growing awareness about AD, leading to an increasing number of individuals expressing concerns about a reduction in their cognition function (Jessen et al., 2020). Furthermore, individuals with personal exposure to dementia may develop heightened sensitivity to specific signs of memory loss (Lee et al., 2021). In this respect, it has been suggested that individuals who express concerns about perceived decline in cognitive function have an increased risk of developing cognitive decline or dementia (Jessen et al., 2020).

Although the progression of dementia is unstoppable because there is not yet a definitive treatment available, certain risk and protective factors associated with dementia are potentially modifiable (Livingston et al., 2020; Ramos et al., 2021b). It is possible to reduce the risk through specific lifestyle changes, delay the onset or slowing down the progression of the disease (World Health Organization, 2019). In this regard, the sooner a patient with cognitive dysfunction is identified, the earlier an appropriate intervention can be carried out to control risk factors and promote a healthy lifestyle. For this reason, screening for CI should be established early to prevent its development at later ages (World Health Organization, 2012).

Up-to-date research knowledge and dissemination of information about modifiable risk factors are crucial to promote effective prevention programs (Rosenberg et al., 2018). In addition, it has been reported that the development and greater accessibility of valuable tools and training would better equip community pharmacists to use their existing knowledge and improve their comfort in managing patients with or at risk of dementia (Chong et al., 2021).

With this purpose in mind, the *A-to-Z Dementia Knowledge list* was elaborated to facilitate memorizing factors associated with dementia (Ramos et al., 2021b). In addition to the clear evidence for the usefulness of 12 factors reported by the Lancet Commission, Alzheimer's Disease International (ADI), and the World Health Organization (WHO) (Morley et al., 2015; Prince et al., 2016; World Health Organization, 2017; Livingston et al., 2020), the *A-to-Z Dementia Knowledge list* includes five more significant factors forming an alphabet and make them easier to remember. To better understand the factors associated with dementia, they are classified according to their influence on cognitive dysfunction into non-modifiable factors (age, sex, genetic), factors that are difficult to modify (education level, job), protective factors (healthy habits such as exercise or good nutrition, cognitive stimulation such as quizzes and mind games, surfing on the internet, reading, meeting friends or playing music to keep mentally active, patient's knowledge of dementia) and risk factors (diseases such as depression, hypertension, insulin resistance, lipid profile alterations, brain injuries, hearing loss, obesity or viral and bacterial infections, memory complaint, environmental exposure to pollution, use of certain pharmaceuticals like anticholinergic drugs or benzodiazepines, toxic habits such as smoking and alcohol consumption, poor sleep hygiene) (Ramos et al., 2021b).

Psychosocial variables are major contributors to cognitive decline and general health status and should be considered as relevant as other biological variables in healthy aging and dementia (Deaton and Stone, 2015). The joint assessment of the influence of psychosocial, clinical, and lifestyle-related variables provides relevant information for the CI course analysis. These include physical activity, nutrition, social interaction, and occupation (García et al., 2022).

The main purpose of this study was to measure the influence of factors included in the *A-to-Z Dementia Knowledge list* in patients at risk of CI concerned about their cognition who were screened in healthcare facilities (including Community Pharmacy, Primary Care Health Centre, and Hospital).

## 2. Materials and methods

### 2.1. Type of study and target population

A cross-sectional observational study was conducted to estimate whether patients were at CI risk for having obtained in the CI assessment a score compatible with CI within a 24-month timeframe and whether it was related to risk and protective factors associated with dementia according to the *A-to-Z Dementia Knowledge*. Individuals with at least one test result compatible with CI were referred to primary care for evaluation as patients with CI after cognitive assessment were considered to have an increased risk of developing dementia.

**Figure 1:**
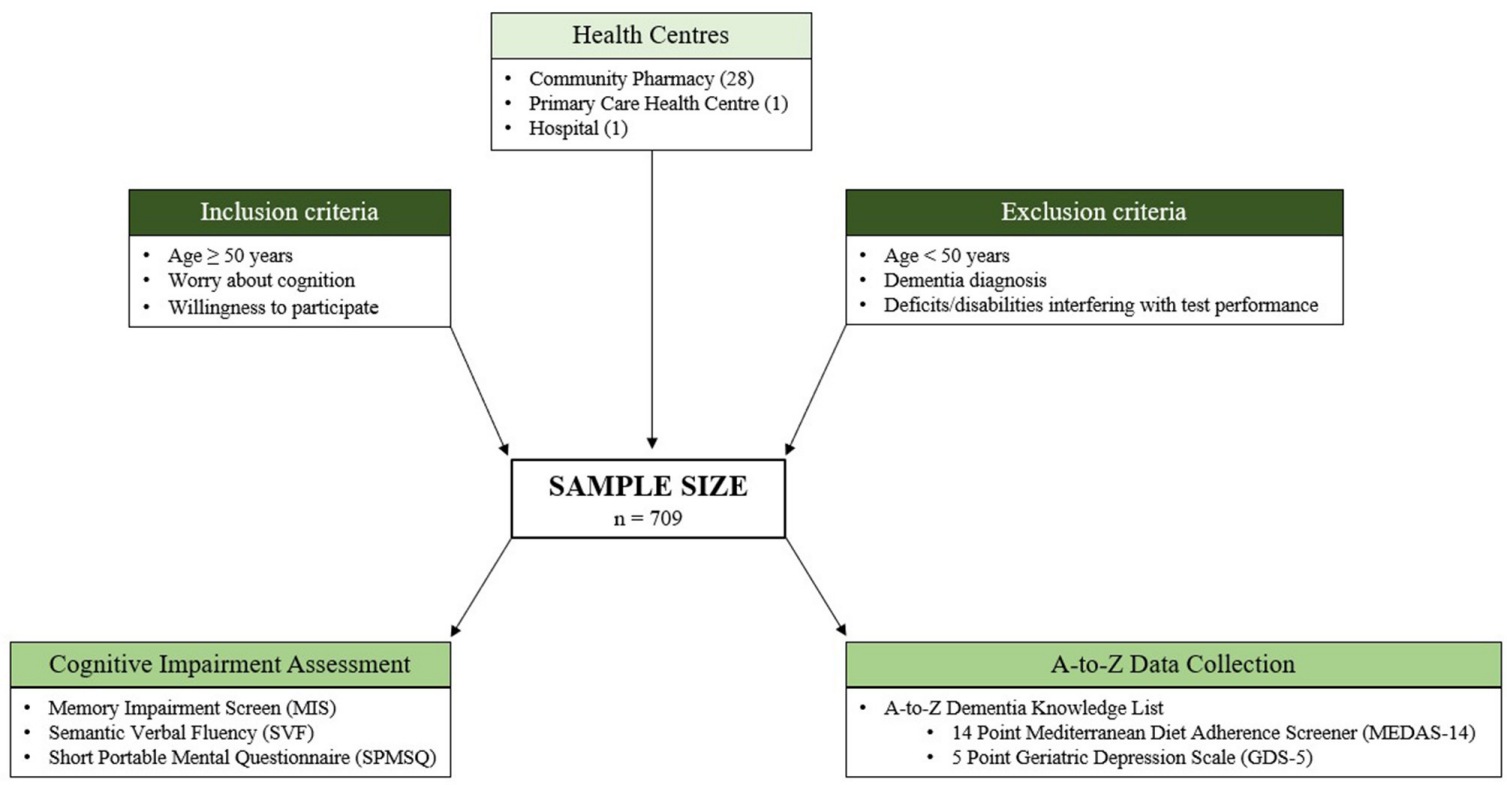
Diagram showing our inclusion criteria and analysis methodology.

As summarized in Figure 1, the following validated CI screening tests were carried out: Memory Impairment Screen (MIS) (Böhm et al., 2005), Semantic Verbal Fluency (SVF) (López Pérez-Díaz et al., 2013), and Short Portable Mental State Questionnaire (SPMSQ) (Martínez de la Iglesia et al., 2001). Using tests with different sensitivity and specificity is essential to obtain diagnostic accuracy. In order to gather information about factors associated with dementia, the interview included additional lifestyle variables and dietary habits and two more screening tests: Mediterranean Diet Adherence Screener (MEDAS-14) (Ferreira-Pêgo et al., 2016) and Geriatric Depression Scale (GDS-5) (Ortega Orcos et al., 2007).

The inclusion criteria, defining the target population were age 50 or older, worried about their cognition, and willingness to participate. Conversely, exclusion criteria were diagnosis of dementia, severe sensory deficits such as blindness or deafness, and physical disability interfering with the performance of the tests. The inclusion age (50 years or older) was decided to detect patients in the early stages of CI (Climent et al., 2018).

The service was offered to regular participating healthcare facility patients (28 Community Pharmacies, 1 Primary Care Health Centre, and 1 Hospital) who met the selection criteria. Likewise, patients directly referred by their physician were included.

### 2.2. Cognitive impairment assessment

#### 2.2.1. Memory impairment screen

The MIS is a short 4-item test that measures the free and selectively facilitated recall, scoring on a 0–8 range. It uses the techniques of controlled learning and selectively facilitated recall to optimize encoding processes. The accepted cut-off point is ≤4 points, in which the sensitivity shown for dementia in the Spanish population was 80%, with a specificity of 96% (Buschke et al., 1999). Therefore, the MIS is proper as a screening instrument for memory problems such as cognitive impairment. In a blinded study, it showed a sensibility of 91.9 (IC95% 83.4–96.4) and a specificity of 81% (IC95% 70.3–88.6). Moreover, this questionnaire also has a sensitivity and specificity for AD, the most common cause of CI, that ranges from 86 to 96%, respectively (Buschke et al., 1999; Böhm et al., 2005).

#### 2.2.2. Semantic verbal fluency

The SVF questionnaire assesses the number of items of a specific category (e.g., animals) within a limited time (1 min). This questionnaire is easy and fast to apply and is very sensitive (74%) and specific (80%) for cognitive impairment (López Pérez-Díaz et al., 2013), which justifies its use for the detection of CI with a cut-off point of fewer than 10 points. Furthermore, as it is a very specific questionnaire for temporal lesions, it is widely used in patients with amnesic mild cognitive impairment, where there is a progressive loss of semantic memory due to alterations in the frontal and temporal lobes (Price et al., 2012; López Pérez-Díaz et al., 2013).

#### 2.2.3. Pfeiffer's short portable mental STATE questionnaire

The SPMSQ assesses different intellectual aspects, including short-term memory, long-term memory, orientation to surroundings, information about recent events, and the ability to perform serial mathematical tasks (Pfeiffer and Short Portable Mental, 1975). This questionnaire is characterized by its brevity and portability, as it assesses ten simple items and it presents a cut-off point of 3 or more errors. The Spanish version of this test obtained a sensitivity of 85.7% and a specificity of 79.3%, respectively (Martínez de la Iglesia et al., 2001).

Participants were considered cognitively impaired if they tested positive for at least one of these tests.

### 2.3. A-to-Z data collection/information collection questionnaires

#### 2.3.1. A-to-Z dementia knowledge list

A data collection booklet was used to gather information on all factors covered in the *A-to-Z Dementia Knowledge List* (Table 1) (Ramos et al., 2021b).

**Table 1:** Questions included in the A-to-Z booklet according to the factors' classification.

**Alphabet letter**	**A-to-Z Factor**	**Type of factor**	**Data collection booklet**
A	Audition	Risk	Hearing loss YES/NO
B	Brain injury	Risk	Brain injury YES/NO
C	Complaint of memory	Risk	Complaint of memory YES/NO
D	Depression	Risk	Depression diagnosis YES/NO GDS-5 result compatible with depression YES/NO
E	Exercise	Protective	Hours of exercise/week
F	Friends	Protective	Do you feel alone? YES/NO Do you feel lonely? YES/NO N°. of friends met the last week
G	Genetics	Non-modifiable	Family history of dementia YES/NO
H	Hypertension	Risk	Hypertension diagnosis YES/NO Hypertension treatment YES/NO
I	Insulin resistance	Risk	Diabetes diagnosis YES/NO Diabetes treatment YES/NO
J	Job	Difficult to modify	Occupation
K	Knowledge	Protective	
L	Lipid profile alteration	Risk	Hipercolesterolemia diagnosis YES/NO Hipercolesterolemia treatment YES/NO
M	Musician	Protective	Plays a musical instrument YES/NO Hours/week playing a musical instrument
N	Nutrition	Protective	MEDAS-14 result
O	Obesity	Risk	BMI
P	Pharmaceutical drugs	Risk	Benzodiazepines consumption YES/NO Benzodiazepines use: Insomnia YES/NO Benzodiazepines use: Anxiety YES/NO Anticholinergic consumption YES/NO Anticholinergic burden (ACB Scale) Antiinflamatory consumption YES/NO Antidepressants consumption YES/NO
Q	Quiz	Protective	Memory training YES/NO
R	Reading	Protective	Reading habit Hours reading/week
S	Sleep	Risk	Hours reading/day
T	Toxics	Risk	Smoker/Nonsmoker/Former smoker Smoker: N°. of cigarettes/day Smoking cessation: How many years ago? N°. of alcohol cups/week
U	Universal task	Protective	
V	Virus and infections	Risk	In HSV treatment YES/NO
W	Web	Protective	Internet use YES/NO Hours of internet use/week
X	Xx	Non-modifiable	Woman YES/NO
Y	Your cognitive reserve	Difficult to modify	
Z	Zip code	Risk	Zip code (urban/rural)

GDS-5, 5 Point Geriatric Depression Scale; MEDAS-14, 14 Point Mediterranean Diet Adherence Screener; BMI, Body Mass Index; HSV, Herpes Virus Simplex.

Regarding the job factor, the categorization of occupations by social class was based on the Spanish Society of Epidemiology classification (Regidor, 2001). Additionally, to classify postcodes according to urban or rural areas, we use the criteria of the Ministry of Agriculture, Fisheries and Food of the Spanish Government, according to which “rural areas are defined as the geographical space formed by the aggregation of municipalities with a population of fewer than 30,000 inhabitants and a density of fewer than 100 inhabitants per km^2^” (Ministerio de Agricultura, 2021).

In addition, MEDAS-14 and GDS-15 were used for nutrition and depression factors, respectively, to provide objective data.

The MEDAS was developed to assess compliance with the nutritional intervention of the *Prevención con Dieta Mediterránea* (PREDIMED) study, a multicenter clinical trial aimed at assessing the effects of the Mediterranean diet on the prevention of cardiovascular disease (Schröder et al., 2011). This questionnaire was validated in the Spanish population (Schröder et al., 2011) and recently in other countries such as Germany (Hebestreit et al., 2017). A face-to-face interview adequately classifies individuals according to their PREDIMED score by means of 14 simple response questions—“yes” or “no”—and allows the quality of the entire dietary pattern to be considered. It offers a score from 0 to 14 points (the higher the score, the better the adherence). On the other hand, GDS-5 is the short version of GDS-30 and quantifies depressive symptoms in older adults through 5 questions. It has a maximum score of 5 points and a cut-off point 2. The Spanish version obtained a sensitivity of 82% and a specificity of 98% in a population over 64 years (Ortega Orcos et al., 2007).

### 2.4. Statistical treatment

The information collected from the participants was stored in a Microsoft Excel spreadsheet designed for the study. After the data purification phase, we proceed with the statistical treatment using the advanced statistical software R. First, the categories of the qualitative variables are described with the sample size as the total and available percentages [n (% total, % available)]; that is, without considering and considering missing data, respectively. Quantitative variables are described with the mean and standard deviation (mean ± SD). The association of each qualitative factor from the A-to-Z Dementia Knowledge list with the CI is analyzed with the Chi-square or the Fisher tests. The association of the quantitative variables with the CI is studied with the T-test for independent samples. Finally, the association of the quantitative variables with the number of positive tests of CI is studied with the Kruskal Wallis test. The significance level is indicated with the following code ^*^: *p*-value < 0.05; ^**^: *p*-value < 0.01; ^***^: *p*-value < 0.001.

### 2.5. Ethical considerations

Information processing guarantees both the protection of the data and its security. These data were treated confidentially and lawfully and were used for the purpose for which the respondent had been informed. Thus, this work complied with the European General Data Protection Regulation (RGPD) and Organic Law 3/2018 on the Protection of Personal Data and the Guarantee of Digital Rights. Furthermore, the study complied with the basic principles of the Declaration of Helsinki: respect for the individual (Article 8) and recognition of their right to self-determination and their right to make informed decisions (informed consent, contained in Articles 20, 21, and 22), including participation in research, both at its beginning and throughout the work. The study was reviewed and approved by the Institutional Review Board (IRB) of Universidad CEU Cardenal Herrera (CEII18/027) and by the Research Ethics Committee of Arnau de Vilanova Hospital (CEIm 7/2022). All subjects gave written informed consent following the Declaration of Helsinki.

## 3. Results

After data collection, information is available from a sample of 709 patients. These patients range in age from 50 to 94 years (69.3 ± 10.3). Of them, 523 are female (73.8%), representing the general population of patients over 50 years of age who come to healthcare facilities with concerns about their cognition.

**Figure 2:**
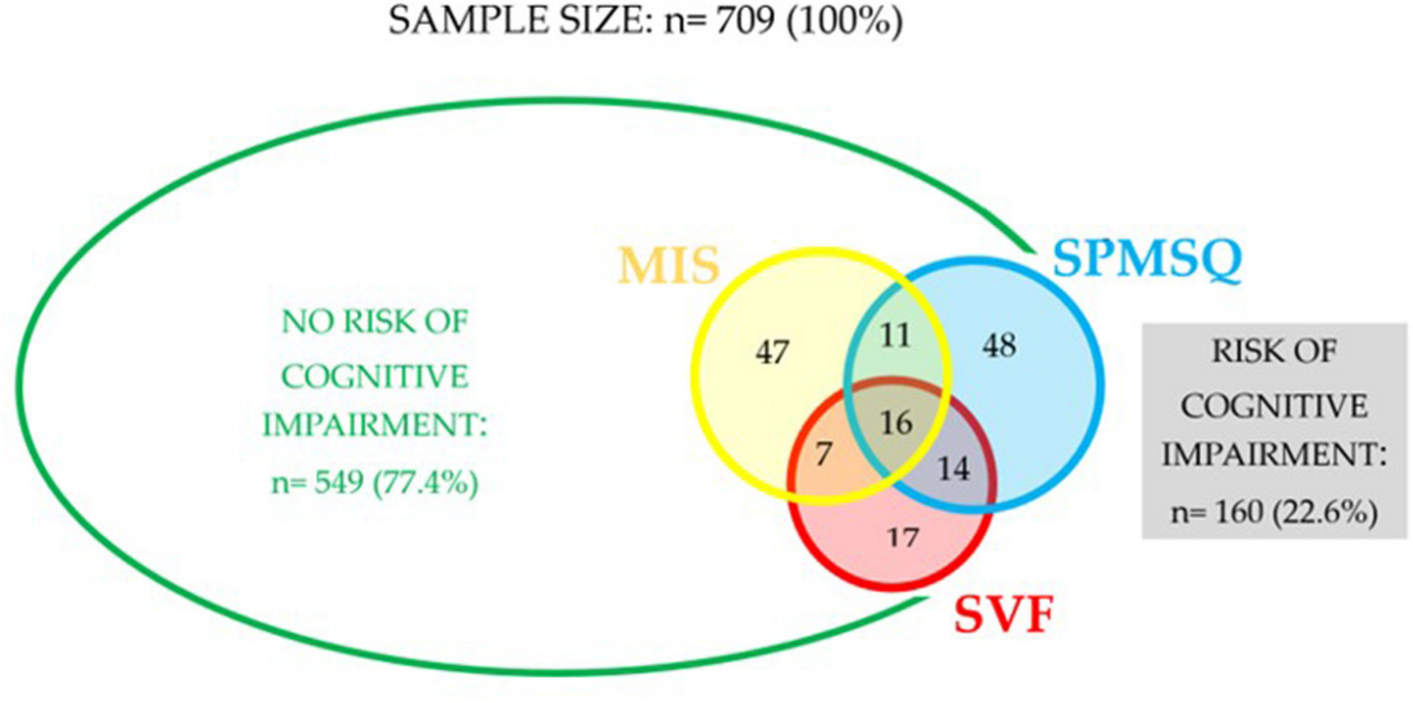
Scheme on the distribution of participating patients between risk and non-risk of cognitive impairment (MIS, Memory Impairment Screen; SPMSQ, Short Portable Mental Status Questionnaire; SVF, Semantic Verbal Fluency).

As shown in Figure 2, after CI screening, according to the three tests mentioned in the methodology (MIS, SVF, and SPMSQ), 160 patients were detected with at least one positive test, and therefore, at risk for CI (22.6%). Concretely, 16 of these patients have all three positive tests (2.3%), 32 have two positive tests (4.5%) and, 112 have a single positive test (15.8%).

Table 2 describes the distribution of patients in the groups with and without risk of CI by age range. As can be seen, patients older than 65 accumulate more than expected in the group with CI, contrary to younger patients.

**Table 2:** Distribution of patients by age range in groups with and without CI (*p*-value of Chi-square test).

	**Totals *n* (% total) 709 (100)**	**Cognitive impairment**		***p*-value**
		**No**	**Yes**	
		**549 (100)**	**160 (100)**	
**Age**				
(50, 65)	236 (33.2)	220 (40.1)	16 (10.0)	< 0.001^***^
(65, 80)	340 (48.0)	258 (47.0)	82 (51.2)	
(80, 94)	133 (18.8)	71 (12.9)	62 (38.8)	

^***^p-value < 0.001.

Although Table 3 analyses the association of all the A-to-Z factors concerning having or not having CI, the text only details those factors that have obtained a statistically significant association and can be modified to reduce CI risk. Qualitative variables are described with the sample size and percentage, n (%), while quantitative variables are described with the mean and standard deviation (mean ± SD).

**Table 3:** Association of the A-to-Z factors vs. having or not having CI.

			**Totals *n* (% total; % available) 709 (100; 100)**	**CI risk**		***p*-value**
				**No**	**Yes**	
				**549 (100)**	**160 (100)**	
Audition (Hearing loss)		No	464 (65.4; 65.4)	356 (64.8)	108 (67.5)	0.572^a^
		Yes	245 (34.6; 34.6)	193 (35.2)	52 (32.5)	
Brain injury		No	681 (96.1; 96.5)	528 (96.4)	153 (96.0)	1.000^c^
		Yes	25 (3.5; 3.5)	20 (3.6)	5 (3.2)	
		Missing	3 (0.4; —)	—	—	
Complaint of memory		No	88 (12.4; 12.4)	75 (13.7)	13 (8.1)	0.076^c^
		Yes	621 (87.6; 87.6)	474 (86.3)	147 (91.9)	
Depression	Diagnosis	No	519 (73.2; 73.3)	403 (73.5)	116 (72.5)	0.839^a^
		Yes	189 (26.7; 26.7)	145 (26.5)	44 (27.5)	
		Missing	1 (0.1; —)	—	—	
	Risk (GDS-5)	No	479 (67.6; 70.9)	390 (73.9)	89 (60.1)	**0.001** ^a**^
		Yes	197 (27.8; 29.1)	138 (26.1)	59 (39.9)	
		Missing	33 (4.7; —)	—	—	
Exercise		No	147 (20.7; 21.3)	106 (19.9)	41 (26.0)	0.180^a^
		< 7 h	313 (44.1, 45.4)	250 (46.8)	63 (40.4)	
		>7 h	230 (32.4, 33.3)	178 (33.3)	52 (33.3)	
		Missing	19 (2.7; —)			
		Hours/week	7.3 ± 7.0	7.1 ± 6.9	7.8 ± 7.1	0.344^b^
Friends	Lives alone	No	561 (79.1; 79.1)	449 (81.8)	112 (70.0)	**0.002** ^a**^
		Yes	148 (20.9; 20.9)	100 (18.2)	48 (30.0)	0.200^a^
	Feels lonely (Lives alone)	No	60 (8.5, 60.6)	42 (65.6)	18 (51.4)	0.130^a^
		Yes	39 (5.5; 39.4)	22 (34.4)	17 (48.6)	
		Missing	49 (6.9; —)	—	—	
	N°. of friends met last week (Lives alone)		2.6 ± 4.3	3.1 ± 5.0	1.6 ± 2.1	0.065^b^
Genetics		No	463 (65.3; 65.5)	340 (62.0)	123 (77.4)	**< 0.001** ^a***^
		Yes	244 (34.4; 34.5)	208 (38.0)	36 (22.6)	
		Missing	2 (0.3; —)	—	—	
Hypertension	Diagnosis	No	346 (48.8; 49.0)	287 (52.4)	59 (37.3)	**0.001** ^a**^
		Yes	360 (50.8; 51.0)	261 (47.6)	99 (62.7)	
		Missing	3 (0.4; —)	—	—	
	Treatment in those who are diagnosed	No	17 (2.4; 4.7)	15 (5.7)	2 (2.0	0.171^c^
		Yes	343 (48.4; 95.3)	246 (94.3)	97 (98.0)	
		Missing	3 (0.4; —)	—	—	
Insulin resistance	Diagnosis	No	561 (79.1; 79.3)	450 (82.1)	111 (69.8)	**0.001** ^a**^
		Yes	146 (20.6, 20.7)	98 (17.9)	48 (30.2)	
		Missing	2 (0.3; —)			
	Treatment in those who are diagnosed	No	4 (0.6; 2.7)	3 (3.1)	1 (2.1)	1.000^c^
		Yes	142 (20.0; 97.3)	95 (96.9)	47 (97.9)	
		Missing	2 (0.3; —)	—	—	
				**No**	**Yes**	
				**549 (100)**	**160 (100)**	
Job		Level 1	54 (7.6; 7.8)	47 (8.8)	7	**< 0.001** ^c***^
		Level 2	110 (15.5,15.9)	93 (17.4)	17 (10.8)	
		Level 3	108 (15.2; 15.6)	89 (16.6)	19 (12.0)	
		Level 4	129 (18.2; 18.6)	96 (17.9)	33 (20.9)	
		Level 5	87 (12.3; 12.6)	72 (13.5)	15 (9.5)	
		Level 6	199 (28.1; 28.7)	132 (24.7)	67 (42.4)	
		Missing	22 (3.1; —)	—	—	
Lipid profile	Diagnosis	No	380 (53.6; 53.6)	303 (55.2)	77 (48.1)	0.126^a^
		Yes	329 (46.4; 46.4)	246 (44.8)	83 (51.9)	
	Treatment in those who are diagnosed	No	24 (3.4; 3.4)	20 (8.1)	4 (4.8)	0.464^c^
		Yes	305 (43.0; 43.0)	226 (91.9)	79 (95.2)	
Musician		No	668 (94.2; 94.2)	513 (93.4)	155 (96.9)	0.124^c^
		Yes	41 (5.8; 5.8)	36 (6.6)	5 (3.1)	
		Hours/week	8.4 ± 7.6	8.8 ± 7.5	5.9 ± 8.9	0.437^b^
Nutrition (MEDAS-14)		Low	71 (10.0; 12.1)	52 (11.3)	19 (15.0)	0.527^a^
		Intermediate	374 (52.8; 63.5)	296 (64.1)	78 (61.4)	
		High	144 (20.3; 24.4)	114 (24.7)	30 (23.6)	
		Missing	120 (16.9; —)	—	—	
Obesity (BMI)		Insufficient	11 (1.6; 1.6)	7 (1.3)	4 (2	0.705^c^
		Normal	189 (26.7; 27.9)	146 (27.9)	43 (27.7)	
		Overweight	271 (38.1; 39.8)	211 (40.2)	60 (38.7)	
		Obese	207 (29.3; 30.7)	159 (30.6)	48 (31.0)	
		Missing	31 (4.4; —)	—	—	
		Score	27.5 ± 4.6	27.5 ± 4.6	27.5 ± 4.7	0.981^b^
Pharmaceutical drugs	Benzodiazepines	No	474 (66.9; 67.0)	378 (69.0)	96 (60.4	**0.045** ^a*^
		Yes	233 (32.9; 33.0)	170 (31.0)	63 (39.6)	
		Missing	2 (0.3; —)	—	—	
	Anticholinergic	No	559 (78.8; 81.6)	436 (81.6)	123 (81.5)	1.000^c^
		Yes	126 (17.8; 18.4)	98 (18.4)	28 (18.5)	
		Missing	24 (3.4; —)	—	—	
		ACB score	1.8 ± 1.1	1.7 ± 1.1	2.0 ± 1.1	0.214^b^
	Antiinflamatories	No	559 (78.8; 79.5)	433 (79.4)	126 (79.7)	1.000^c^
		Yes	144 (20.3; 20.5)	112 (20.6)	63 (39.6)	
		Missing	6 (0.8; —)	—	—	
	Antidepressants	No	540 (76.2; 76.3)	417 (76.1)	123 (76.9)	0.916^a^
		Yes	168 (23.7; 23.7)	131 (23.9)	37 (23.1)	
		Missing	1 (0.1; —)	—	—	
				**No**	**Yes**	
				**549 (100)**	**160 (100)**	
Quiz (Memory training)		No	364 (51.3; 51.6)	269 (49.2)	95 (59.7)	**0.019** ^a*^
		Yes	342 (48.2; 48.4)	278 (50.8)	64 (40.3)	
		Missing	3 (0.4; —)	—	—	
Reading		No	227 (32.0; 32.0)	151 (27.5)	76 (47.5	**< 0.001** ^a***^
		Yes	482 (68.0; 68.0)	398 (72.5)	84 (52.5)	
		Hours/week	6.4 ± 7.5	6.5 ± 7.5	6.2 ± 8.0	0.801^b^
Sleep		< 6 h	112 (15.8; 17.7)	87 (17.8)	25 (17.6)	**0.013** ^c*^
		(6–9 h)	486 (68.5; 76.9)	384 (78.4)	102 (71.8)	
		< 9 h	34 (4.8; 5.4)	19 (3.9)	15 (10.6)	
		Missing	77(10.9; —)	—	—	
		Hours/day	7.0 ± 1.6	6.8 ± 1.5	7.3 ± 2.1	**0.002** ^b**^
Toxics	Tobacco	Non smoker	388 (54.7; 54.8)	278 (50.6)	110 (69.2	**< 0.001** ^c***^
		Former smoker	236 (33.3; 33.3)	198 (36.1)	38 (23.9)	
		Smoker	84 (11.8; 11.9)	73 (13.3)	11 (6.9)	
		Missing	1 (0.1; —)	—	—	
		Years without smoking (Former smoker)	21.3 ± 12.5	21.0 ± 12.0	22.6 ± 14.9	0.483^b^
		Cigarettes/day (Smoker)	12.4 ± 9.0	11.8 ± 7.8	16.1 ± 14.0	0.141^b^
	Alcohol	No	260 (36.7; 55.0)	199 (52.6)	61 (64.2)	0.050^c^
		Yes	213 (30.0; 45.0)	179 (47.4)	34 (35.8)	
		Missing	236 (33.3; —)	—	—	
		Cups/week (Those who drink)	1.6 ± 2.7	1.6 ± 2.7	1.6 ± 2.7	0.891^b^
Universal task (Useless feeling)		No	239 (33.7; 83.6)	174 (84.1)	65 (82.3	0.723^c^
		Yes	47 (6.6; 16.4)	33 (15.9)	14 (17.7)	
		Missing	423 (59.7; —)	—	—	
Virus or infection	HVS diagnosis	No	527 (74.3; 74.8)	392 (71.9)	135 (84.4)	**0.001** ^c**^
		Yes	178 (25.1; 25.2)	153 (28.1)	25 (15.6)	
		Missing	4 (0.6; —)	—	—	
	Treatment in those who are diagnosed	No	47 (6.6; 28.0)	37 (25.7)	10 (41.7)	0.139^c^
		Yes	121 (17.1; 72.0)	107 (74.3)	14 (58.3)	
		Missing	14 (2.0; —)	—	—	
Web (Internet use)		No	208 (29.3; 29.4)	113 (20.6)	95 (59.7)	**< 0.001** ^a***^
		Yes	500 (70.5; 70.6)	436 (79.4)	64 (40.3)	
		Missing	1 (0.1; —)	—	—	
		Hours/week	10.0 ± 10.7	10.0 ± 11.1	9.5 ± 7.9	0.701^b^
XX (Woman)		No	186 (26.2; 26.2)	139 (25.3)	47 (29.4)	0.309^a^
				**No**	**Yes**	
				**549 (100)**	**160 (100)**	
		Yes	523 (73.8; 73.8)	410 (74.7)	113 (70.6)	
Zip code		Rural	129 (18.2; 21.3)	101 (22.0)	28 (18.9)	0.488^a^
		Urban	478 (67.4; 78.7)	358 (78.0)	120 (81.1)	
		Missing	102 (14.4; —)	—	—	

Numerical results are described as means and standard deviations (mean ± SD) while qualitative results are described with sample sizes and percentages [n (%)]; a: Chi-square test; b: T-test for comparison of two independent means (one-sided); c: Fisher exact test; MEDAS-14: 14 Point Mediterranean Diet Adherence Screener; BMI: Body Mass Index; HVS: Herpes Virus Simplex.

^*^p-value < 0.05.

^**^p-value < 0.01.

^***^p-value < 0.001.

Statistical significance marked in bold.

For example, the mean number of months with hearing loss is significantly lower among those with CI (32.9 ± 49.7) vs. those without CI (64.1 ± 105.9).

GDS-5 determines a statistically significant association in patients at risk of depression: the group with CI is higher than those without CI (39.9 vs. 26.1%).

The percentage of patients living alone is significantly higher in the group with CI than in the group without CI (30 vs. 18.2%). Therefore, living alone is significantly associated with being at risk of CI.

Among patients with a family history of AD, there is a significantly higher percentage in the group without CI compared with the group with CI (38 vs. 22.6%). Not having a family history of AD is significantly associated with having CI.

The percentage of patients with hypertension in the group with CI is also significantly higher compared with the group without CI (62.7 vs. 47.6%). According to this result, having hypertension is significantly associated with having CI. Likewise, the number of patients who have diabetes is significantly higher in the group with CI compared with the group without CI (30.2 vs. 17.9%). Thus, having diabetes is also significantly associated with having CI.

Regarding the occupation role in CI, Level 4 (skilled manual worker) and Level 6 (unskilled manual worker) are observed more than expected in the group with CI. However, occupations Level 1 (professions associated with second and third-cycle university degrees), Level 2 (professions associated with a first-cycle university degree), Level 3 (unskilled non-manual worker and self-employed worker), and Level 5 (semi-skilled manual worker) are observed more than expected in the group without CI. We can observe that the type of occupation performed is associated with CI.

Since the percentage of patients taking benzodiazepines is significantly higher in the group with CI compared with the group without CI (39.6 vs. 31%), benzodiazepine use is significantly associated with having CI.

There are significantly more patients who routinely train their memory in the group without CI compared with the group with CI (50.8 vs. 40.3%). Hence, lack of memory training is significantly associated with having CI. The same applies to patients who read regularly (72.5 vs. 52.5%).

Sleeping more than 9 h per day is significantly higher in the group with CI than the group without CI (10.6 vs. 3.9%). Oversleeping is significantly associated with having CI. In addition, the mean sleep time in the group with CI is significantly higher than in the group without CI (7.3 ± 2.1 vs. 6.8 ± 1.5).

Former smokers and smokers are observed more than expected in the group without CI. However, non-smokers are observed more than expected in the group with CI. Therefore, tobacco exposure is associated with not having CI.

The percentage with a diagnosis of HSV is significantly higher in the group without CI compared with the group with CI (28.1 vs. 15.6%). In this regard, a diagnosis of HSV is significantly associated with not having CI.

More patients regularly use the internet. As a result, it is significantly higher in the group without CI risk compared with the group with CI risk (79.4 vs. 40.3%). Consequently, not using the internet is significantly associated with CI risk.

The association of the A-to-Z factors with the number of positive CI tests was also analyzed to identify trends. Table 4 summarizes the A-to-Z factors that have shown statistically significant associations and can be modified to reduce CI risk.

**Table 4:** Association of the A-to-Z factors vs. the number of positive tests for CI.

		**Number of positive tests**				
		**0**	**1**	**2**	**3**	***p*** **-value**
		***n** =* **549**	***n** =* **112**	***n** =* **32**	***n** =* **16**	
Depression (GDS-5)	No	390 (73.9)	66 (64.1)	14 (46.7)	9 (60.0)	0.003^a**^
	Yes	138 (26.1)	37 (35.9)	16 (53.3)	6 (40.0)	
Do you live alone	No	449 (81.8)	82 (73.2)	21 (65.6)	9 (56.2)	0.004^a**^
	Yes	100 (18.2)	30 (26.8)	11 (34.4)	7 (43.8)	
Hypertension	No	287 (52.4)	40 (36.4)	15 (46.9)	4 (25.0)	0.004^a**^
	Yes	261 (47.6)	70 (63.6)	17 (53.1)	12 (75.0)	
Insulin resistance	No	450 (82.1)	75 (67.6)	23 (71.9)	13 (81.2)	0.004^a**^
	Yes	98 (17.9)	36 (32.4)	9 (28.1)	3 (18.8)	
Job	Intellectual work (Levels 1, 2, 3)	229 (43.3)	36 (32.4)	4 (12.9)	3 (18.8)	0.001^a**^
	Manual work (Levels 4, 5, 6)	300 (56.7)	75 (67.6)	27 (87.1)	13 (81.2)	
Obesity	Insufficient	7 (1.3)	1 (0.9)	3 (10.3)	0 (0.0)	0.036^a*^
	Normal	146 (27.9)	30 (27.3)	7 (24.1)	6 (37.5)	
	Obese	159 (30.4)	37 (33.6)	9 (31.0)	2 (12.5)	
	Overweight	211 (40.3)	42 (38.2)	10 (34.5)	8 (50.0)	
Reading	No	151 (27.5)	49 (43.8)	16 (50.0)	11 (68.8)	< 0.001^a***^
	Yes	398 (72.5)	63 (56.2)	16 (50.0)	5 (31.2)	
Web	No	113 (20.6)	57 (50.9)	24 (77.4)	14 (87.5)	< 0.001^a***^
	Yes	436 (79.4)	55 (49.1)	7 (22.6)	2 (12.5)	
Depression score (GDS-5)		1.1 ± 1.3	1.3 ± 1.4	1.8 ± 1.3	1.6 ± 1.2	0.008^b**^
Nutrition score (MEDAS-14)		9.0 ± 2.1	8.8 ± 2.3	7.6 ± 2.9	8.9 ± 2.2	0.039^b*^
Sleep (hours/week)		6.8 ± 1.5	7.2 ± 2.3	7.6 ± 1.7	7.6 ± 1.4	0.012^b^^*^

Numerical results are described as means and standard deviations (mean ± SD), while qualitative results are described with sample sizes and percentages [n (%)]; CI: cognitive impairment: GDS-5: 5 Point Geriatric Depression ScALE; BMI: Body Mass Index; MEDAS-14: 14 Point Mediterranean Diet Adherence Screener; a: Fisher test; b: Kruskal Wallis test.

^*^p-value < 0.05.

^**^p-value < 0.01.

^***^p-value < 0.001.

For example, the percentage of patients at risk of depression increases significantly as the number of positive CI tests grows. The same applies to the percentage of patients living alone, with a hypertension diagnosis, or with an unskilled manual worker.

Similarly, the percentage of diabetic patients is significantly higher among patients with a positive CI screening test. However, the higher percentage of people with diabetes accumulates in the group with a single positive test rather than among those with more positive tests.

On the other hand, the percentage of patients who read, use the Internet regularly or have intellectual work decreases as the number of positive CI tests increases.

**Figure 3:**
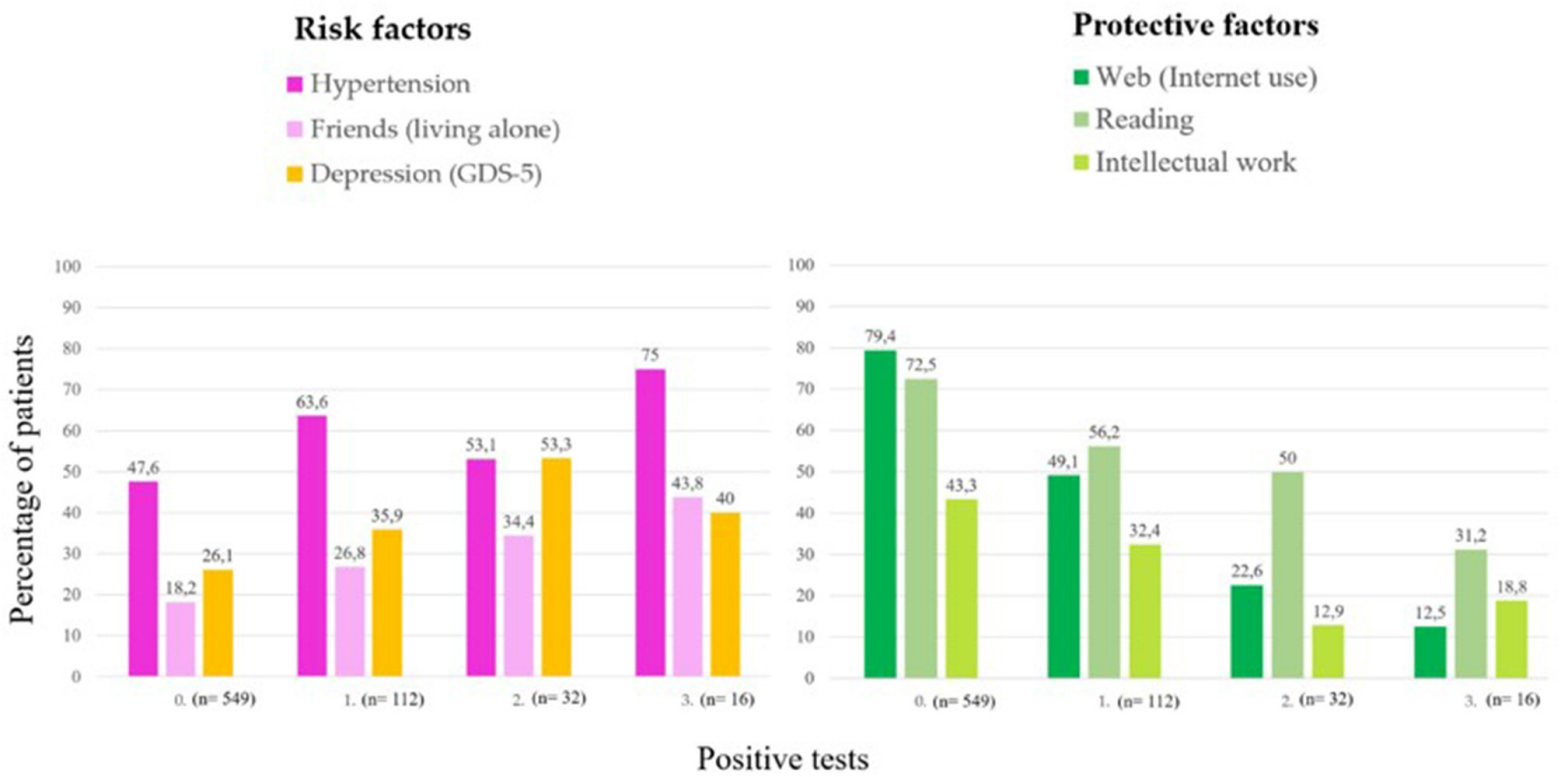
Evolution of the percentage of patients with hypertension, loneliness, depression, Internet use, reading, and intellectual work, as the number of positive CI screening tests increases.

Using the information described in Table 4 for hypertension, loneliness, depression, Internet use, reading, and intellectual work, Figure 3 graphically represents the evolution of the percentages of patients as the number of positive IC tests increases. As can be seen, the first three factors are risk factors for CI since the percentages tend to increase as the number of positive CI tests increases. On the contrary, the other three factors below are protective factors because the tendency of the percentages decreases as the number of positive CI tests increases.

## 4. Discussion

The main contribution of this work is the estimation of factors included in the *A-to-Z Dementia Knowledge list* in a sample of cognitively concerned patients screened for CI. Various factors influence this estimation in addition to regular age-related degenerative changes (Cheon, 2022). Therefore, addressing a combination of modifiable factors is currently suggested to be the best approach for mitigating or preventing the onset of dementia (Iadecola and Parikh, 2020). Our study found that hypertension, loneliness, and depression were gradually associated with cognitive decline as potential risk factors. In contrast, internet use, reading, and type of job were gradually associated with less cognitive decline, suggesting a protective effect (Figure 3).

According to the 2020 report of the Lancet Commission, there are specific potentially modifiable risk factors for dementia. As stated in this report, risk factors during early life, midlife, and later life can contribute to increased risk of dementia, as indicated by the following population attributable fraction (PAFs): less education (7.1%), hearing loss (8.2%), traumatic brain injury (3.4%), hypertension (1.9%), more than 21 units of alcohol/week (0.8%), obesity with BMI ≥ 30 (0.7%), smoking (5.2%), depression (3.9%), social isolation (3.5%), physical inactivity (1.6%), diabetes (1.1%) and air pollution (2.3%) (Morley et al., 2015).

Firstly, factors related to metabolic syndrome are highlighted. These include hypertension, insulin resistance, an altered lipid profile, and obesity. Reducing cardiovascular risk represents one of the most viable and promising strategies, as its association with CI is well known (Farnsworth Von Cederwald et al., 2022). The detrimental effect of vascular risk in mid-life on the future development of dementia has also been highlighted (McGrath et al., 2020). Hypertension is one of the most important risk factors for dementia, as it can be controlled and modified (Cheon, 2022). In addition, long-term cumulative blood pressure has been associated with subsequent cognitive decline and risk of dementia (Li C. et al., 2022). Given the high prevalence of dementia and its impact on quality of life, treating hypertension to reduce CI may be a clinically relevant intervention, Observational and randomized trials have shown that reducing blood pressure is associated with less dementia and CI (Iadecola and Parikh, 2020; Cheon, 2022), suggesting a 7–11% relative risk reduction in the incidence of dementia with antihypertensive treatment (Canavan and O'Donnell, 2022). On the other hand, numerous studies have linked type 2 diabetes with an increased risk of CI and dementia (Fink et al., 2022). Therefore, by reducing the incidence of diabetes, we can also reduce the incidence of dementia in diabetes patients (Fink et al., 2022). Moreover, diabetes mellitus has been identified as one of the risk factors responsible for up to one-third of AD cases and represents an important modifiable target for preventing dementia at the population level (McGrath et al., 2020). Cognitive-behavioral therapy for lifestyle modification in patients with metabolic syndrome effectively reduces cardiovascular risk (Garcia-Silva et al., 2022).

Regarding depression, this condition is closely associated with the incidence of dementia, and there are several potential mechanisms involved. These mechanisms include increased cortisol levels, vascular difficulties, inflammation, decreased brain-derived neurotropic factor, telomere shortening, increased plasma levels of amyloid ß42, and neurofibrillary tangles (Linnemann and Lang, 2020). Different studies have found positive associations between depression and dementia. It remains to be determined whether depression is a prodromal symptom of dementia, a risk factor, or a consequence of cognitive decline. They could also coexist due to a common underlying pathology or similar symptoms in both conditions (Sjöberg et al., 2020). In our study, we observed statistically significant differences in the reported depressive state as measured by GDS-5 but not in the diagnosis of depression itself. It could be because the depressive state directly influences the assessment of depression diagnosis. On the other hand, depression may be underdiagnosed in some patients, or the effectiveness of pharmacological treatment in diagnosed patients could lead to a positive score on the GDS-5.

The main difference between depression and other cognitive risk factors is the availability of various therapeutic options, as some antidepressants may worsen the cognitive impact of depression. Therefore, studies have shown that using social supports, such as reducing social isolation, can delay the onset of dementia (Hakim, 2022). The potential increase in loneliness due to population aging and social isolation may harm brain health (Tao et al., 2022). Although living alone does not necessarily imply social isolation, loneliness feeling, or poor social networks, it is essential to note that social networks tend to diminish in later life due to factors such as adult children becoming independent, the loss of close social contacts through death and increased selectivity of social interactions with age. In addition, late-life implies health deterioration and limited mobility, which can further limit engagement in social activities and reinforce feelings of isolation (Evans et al., 2019). While living alone is an objective observation, loneliness refers to subjective dissatisfaction with social relationships and can be perceived differently by individuals. In line with our results, it has been suggested that living alone in later life may increase the risk of poor cognitive function. From a cognitive reserve perspective, living with others may enhance cognitive stimulation through social interaction, as there are more opportunities for social engagement (Evans et al., 2019). Socially stimulating environments promote neuroprotective mechanisms by activating alternative pre-existing or compensatory cognitive processes (Samtani et al., 2022). Frequent social activity has also been associated with improved memory, executive function, visuospatial ability, and processing speed, whereas frequent social support has been linked to improved memory (Kelly et al., 2017).

Concerning the protective factors gradually associated with reduced CI, certain variables related to cognitive stimulation stand out. These include internet use, reading, and type of job. Given the lack of effective pharmacological treatment, non-pharmacological activities are an important alternative to consider for promoting cognitive stimulation and delaying the onset of dementia (Yu et al., 2022). In this context, the concept of cognitive reserve becomes significant. Cognitive reserve refers to the varying susceptibility to exhibit dementia symptoms during the same phases of the disease (Stern, 2013; Stern and Barulli, 2019; Stern et al., 2021). Cognitive reserve is not immutable but is influenced by different exposures throughout life. These include general cognitive ability in early life, education, occupation, physical exercise, leisure activities, and social engagement (Cheng, 2016). As observed in our study, cognitive stimulation variables such as internet use, reading, quizzes, and mind games are statistically significantly associated with reduced CI. These data are consistent with previous studies, suggesting that modifiable lifestyle factors, like reading and daily Internet use, can slow cognitive decline in patients aged 50 and above with SMC (Ramos et al., 2021a).

Recent findings have also highlighted the interaction between technology, social environment, and cognitive functioning in later life (Kim and Han, 2022). Computerized cognitive training has also recently become a potential cognition stimulation instrument (Li R. et al., 2022). While internet use has shown cognitive benefits, discontinuation of internet use has been found to have adverse effects (Kim and Han, 2022). Different levels of internet use could have different relationships with cognitive function in middle-aged and older adults (Yu et al., 2022). Furthermore, social networking sites can also contribute to social support and connection and reduce perceived social isolation (Yu et al., 2022).

On the other hand, the results obtained regarding reading are in line with previous studies. A longitudinal study with 14 years of follow-up linked reading to a protective effect on cognitive function in late life (Chang et al., 2021). Furthermore, another cross-sectional study revealed that reading, writing, and technology use frequencies were significantly associated with language, attention, and memory proficiency after adjusting for demographic characteristics (Iizuka et al., 2021). In line with these findings, a 6-year follow-up study in Japan associated a lower risk of cognitive decline among individuals who reported being readers, regardless of whether they considered reading a hobby (Sugita et al., 2021). Finally, a mixed-effects model revealed that more frequent and earlier cognitive activity during a 5.8-year follow-up was associated with slower cognitive decline (Wilson and Boyle, 2013). Among the cognitive activities considered reading books, visiting a library, and writing letters were consistent with the cognitive reserve hypothesis.

Regarding the type of work, several studies have found that the risk of dementia is lower in people with cognitively stimulating jobs than those with non-stimulating jobs (Huang et al., 2020; Kivimäki et al., 2021). In a sample of 2261 participants, cognitive stimulation was associated with lower levels of plasma proteins that potentially hinder axonogenesis and synaptogenesis, consequently increasing the risk of dementia (Kivimäki et al., 2021). Moreover, a systematic review and meta-analysis concluded that engaging in mentally challenging work is linked to a reduced risk of MCI. Furthermore, working with more complex data and interacting with people may also decrease the risk of dementia (Huang et al., 2020). However, it is worth noting that job strain may influence cognitive performance decline in (Huang et al., 2020). Therefore, our findings, which show a significant inverse association between intellectual work and CI, align with previous research studies.

There is accumulating evidence linking sleep disturbances to the risk of dementia. Consistent with our findings, prolonged sleep duration (9 h per night) has been associated with an increased risk of late-life dementia (Sindi et al., 2018).

To date, the literature supports that hearing loss is a modifiable risk factor interrelated with dementia, and hearing aids can play a significant role in cognitive health. Both hearing loss and CI include aging, mitochondrial dysfunction, microvascular factors, and inflammation (Tarawneh et al., 2022). Given that mid-life hearing loss precedes the onset of dementia and may may contribute to up to 9.1% of dementia cases worldwide, it should be targeted as a preventive strategy for managing dementia (Ford et al., 2018; Pichora-Fuller, 2020). Although we did not observe statistically significant differences, this could be attributed to our homogeneous sample of health-conscious patients.

Our study did not observe statistically significant differences between memory complaints and CI. Nevertheless, it is worth noting that memory complaint is a variable that may be present in stage 2 of AD (Jessen et al., 2020). This factor has been associated with a twofold increase in the likelihood of dementia (Mitchell et al., 2014). In addition, it has been observed that preclinical AD patients with memory complaints had a 62% higher risk of progression from MCI to dementia within 3 years (Wolfsgruber et al., 2017).

Regarding genetics, statistically significant differences were observed in our study between the absence of family history and CI. Although AD has an estimated heritability of 58–79% in early-onset AD and 90% in late-onset AD, the reality is that purely genetic AD is < 1%, which can be explained by Mendelian inheritance pattern (Van Cauwenberghe et al., 2016; Potter et al., 2020). However, it is known that potentially modifiable risk factors play an important role in this disease, influencing 40% of the risk of dementia (Morley et al., 2015). Therefore, the obtained results could be attributed to patients with a family history having a better understanding of the disease and its associated risk factors.

Although numerous studies have associated anticholinergic drugs with CI (Chatterjee et al., 2020; Pasina et al., 2020; Sargent et al., 2020; Weigand et al., 2020), we did not find a statistically significant association in our study, which aligns with a previous study conducted by our group, where an association between CI and the anticholinergic burden was observed when measured using the newly developed CRIDECO Anticholinergic Load Sclae (CALS), which includes 129 new drugs with anticholinergic effects. However, no association was found when using the currently most widely used anticholinergic scale, the Anticholinergic Burden Scale (ACB). It is important to note that our study collected data before developing the new scale (Ramos et al., 2021b). In contrast, we observed an association between CI and the consumption of benzodiazepines, which is consistent with previous studies (Tapiainen et al., 2018; Baek et al., 2020).

Concerning smoking, despite being a known cardiovascular risk factor and, therefore, a risk factor for dementia, it is also known that nicotine may have a protective role in CI (Dong et al., 2020; Rao et al., 2022). In a recent study, nicotine has been found to prevent stress-induced damage in the hippocampus suggesting a potential neuroprotective role (Dong et al., 2020). Moreover, nicotine has shown promise as a treatment for cognitive deficits caused by traumatic brain injury. It can reverse altered signaling pathways in the brain, involving nicotinic receptors, tyrosine hydroxylase, and dopamine (Rao et al., 2022). Therefore, we hypothesize that in the stage of cognitive decline that patients are at, the long-term risks associated with smoking may not be evident, and we only observe the short-term neuroprotective effects of nicotine.

Treatment with antiherpetic medication has been associated with a decreased risk of dementia (Tzeng et al., 2018). In this 2018 study, antivirals were statistically significant in reducing the risk of dementia, highlighting the importance of treating HSV infection when it manifests. However, our study did not find any association.

Age is widely recognized as the primary risk factor for dementia. According to the Comprehensive Plan for Alzheimer's and other Dementias (2017–2023), the prevalence of this disease is around 0.05% among people aged 40–65 years, 1.07% among those aged 65–69 years; 3.4% in 70–74 years; 6.9% in 75–79 years; 12.1% in 80–84 years; 20.1% in 85–89 years; and 39.2% among those over 90 years. As shown in Table 2, our study population consisted of a higher percentage of individuals with cognitive impairment, as one of the inclusion criteria was a concern for cognition (Ministerio de Sanidad Consumo y Bienestar Social., 2019).

In our sample, among all the factors identified in the scientific literature as risk factors, the following are associated with gradual cognitive deterioration: hypertension, living alone, and depression. On the other hand, scientifically identified protective factors include internet use, daily reading, and intellectual work.

This study has several limitations. We could not collect data on the following factors in our patient's: knowledge and universal task. In addition, the results pertain to the CI risk, reflecting a decline in cognitive function in the patients because we do not have data on the diagnosis of CI by a neurologist. Notably, our sample consisted of homogeneous patients concerned about their memory, which may represent a specific group of patients for screening purposes. Future studies with a prospective approach and patient follow-up are needed.

## 5. Conclusion

This study identified the most influential variables that can be modified to reduce CI risk. Our results suggest that a joint assessment of the influence of psychosocial, clinical, and lifestyle-related factors is needed to develop dementia prevention strategies.

## Data availability statement

The raw data supporting the conclusions of this article will be made available by the authors, without undue reservation.

## Ethics statement

The studies involving human participants were reviewed and approved by Institutional Review Board (IRB) of Universidad CEU Cardenal Herrera (CEII18/027) and by the Research Ethics Committee of Arnau de Vilanova Hospital (CEIm 7/2022). The patients/participants provided their written informed consent to participate in this study.

## Author contributions

Conceptualization: MA and MG-P. Methodology, software, and formal analysis: MA. Validation: MG-P and JS-L. Investigation: MG-P, HR, GG-L, CG, TL, and MS. Resources: JS-L. Data curation: MG-P and CG. Writing—original draft preparation: MG-P and HR. Writing—review and editing and Funding acquisition: JS-L and LM. Supervision and project administration: LM. All authors have read and agreed to the published version of the manuscript.
